# Juvenile nasopharyngeal angiofibroma: analysis of 12 cases and review of the literature

**DOI:** 10.1515/med-2025-1312

**Published:** 2026-05-08

**Authors:** Luigi Cofone, Mauro Palmieri, Ivano Pindinello, Antonio Minni, Marco Artico, Francesco Circosta, Gabriele Santilli, Raffaella Carletti, Cira Tiziana Rosaria Di Gioia, Samanta Taurone

**Affiliations:** Department of Public Health and Infectious Diseases, Sapienza University of Rome, Rome, Italy; Department of Human Neurosciences, Sapienza University of Rome, Rome, Italy; Department of Sense Organs, Faculty of Medicine and Odontology, Sapienza University of Rome, Rome, Italy; Department of Movement, Human and Health Sciences, Division of Health Sciences, University of Rome “Foro Italico”, Rome, Italy; Department of Radiological, Oncological and Pathological Science, Sapienza University of Rome, Rome, Italy

**Keywords:** juvenile nasopharyngeal angiofibroma, Transforming Growth Factor Beta (TGF-*β*), vascular endothelial growth factor (VEGF), Tumor Necrosis Factor Alpha (TNF-*α*)

## Abstract

**Objectives:**

Adolescent males are the main victims of juvenile nasopharyngeal angiofibroma (JNA), an uncommon, benign tumor that is extremely vascular and locally aggressive. Because of its invasive growth and proximity to important anatomical systems, JNA presents major therapeutic hurdles despite its benign classification. Understanding the molecular and histological processes behind juvenile nasopharyngeal angiofibroma (JNA) is the goal of the study.

**Methods:**

12 Nasopharyngeal Angiofibroma (8 males and 4 females, mean age 16.2, range 14–18 years) and 4 control samples (normal tissue samples contained no visible tumor cells; three males and one female) were retrieved from archival paraffin embedded blocks with a sufficient sample size. The expression of TGF-β1, VEGF-A, and TNF-α has been studied. The technique chosen for the immunohistochemical study of the samples was microdensitometry.

**Results:**

By analyzing tissue samples, it investigates the presence of Transforming Growth Factor Beta (TGF-β), Vascular Endothelial Growth Factor (VEGF), Tumor Necrosis Factor Alpha (TNF-α) in angiogenesis, fibrosis, and inflammation—three critical processes in the development of malignancies. Overexpression of these growth factors was found in tumor tissues by immunohistochemical analysis, indicating their role in vascularization and tumor progression.

**Conclusions:**

These findings suggest potential targets for treatment, such as anti-angiogenic drugs, to improve management strategies beyond surgical resection, which remains the primary therapeutic strategy. Future research should focus on developing novel therapy strategies that use molecular inhibitors to improve clinical outcomes for JNA patients.

## Introduction

A rare, benign, but locally aggressive vascular tumor, juvenile nasopharyngeal angiofibroma (JNA) primarily affects male adolescents, usually between the ages of 10 and 25 [1]. Despite being categorized as benign, JNA is often very invasive, and its proximity to vital anatomical structures, including the sinuses, cranial nerves, and the carotid artery, makes identification, management, and treatment extremely difficult [2], [3], [4]. Initial symptoms of the condition are rather nonspecific and usually include a nasopharyngeal mass, nasal obstruction, and/or recurrent unilateral epistaxis [4], [5], [6]. For efficient management and patient care, a comprehensive understanding of JNA is necessary due to its propensity to infiltrate surrounding tissues and its potential for significant morbidity, eroding bone and tissues [3], 5].

Research into the pathophysiology of JNA is still ongoing. Hormonal variables, especially androgens, have been linked to the formation of the tumor, although the exact etiology is yet unknown [7]. It is believed that the tumors originate from the vascular-rich nasopharyngeal mucosa and have a propensity to spread into the orbit, the infratemporal fossa, and the paranasal sinuses. The presence of a vast network of blood vessels that are prone to bleeding is a major factor in its growth, making surgical treatment of the tumor particularly difficult [1], 6].

In clinical terms, JNA typically presents as unilateral nasal obstruction, recurrent epistaxis (nosebleeds), and hearing loss, though facial edema, headaches, or ocular symptoms may occasionally be observed. These ambiguous symptoms often lead to a delay in diagnosis, which prolongs the time it takes for the tumor to be identified. Advanced imaging methods, including magnetic resonance imaging (MRI) and contrast-enhanced computed tomography (CT), are crucial for assessing the tumor’s size, vascularity, and relationship to adjacent tissues to aid in surgical planning and decision-making [1], 8].

JNA is divided into three categories based on radiological and clinical characteristics. Lesions that are primarily localized to the nasal cavity, paranasal sinus, nasopharynx, or pterygopalatine fossa are included in type I. Type II JNAs have complete dura mater but extend anteriorly and/or minimally into the middle cranial fossa, infratemporal fossa, buccal area, or orbital cavity. Type III is a large tumor lobe in the middle cranial fossa that resembles a calabash [9].

The primary treatment option for JNA is surgical resection [10]; however, the tumor’s size, location, and extension into adjacent structures such as a large intracranial extension or encase the optic nerve or internal carotid artery often determine the optimal approach. Surgical excision is challenging due to the tumor’s vascularity and the potential for significant bleeding during the procedure. Preoperative embolization of the tumor’s blood vessels is often performed to minimize intraoperative bleeding and facilitate safer excision [10]. Even with advancements in surgical techniques and adjuvant therapies, including chemotherapy, hormone administration, radiation therapy, and embolization. Recurrence rates remain a concern, particularly in situations where complete excision is not feasible. Therefore, long-term surveillance is necessary to spot any signs of residual growth or tumor recurrence [1], 8].

Novel insights into possible treatment targets have been made possible by recent developments in our understanding of the molecular and genetic foundation of JNA. Relevant chromosomal changes have been identified by recent investigations into the genetic and molecular pathways of JNA. Studies found loss in some areas (chromosomes 17, 22, and Y) and gains in others (chromosomes 4q, 6q, 8q, and X) [11]. It’s interesting to note that a lot of cases had extra copies of the androgen receptor (AR) gene and Y chromosome deletion, which supports the theory that male hormones may contribute to tumor growth. The occurrence of *β*-catenin mutations, primarily in stromal (connective tissue) cells, is the most important discovery. This implies that instead of coming from blood arteries as was previously believed, JNA may come from these stromal cells. It may be because of the interaction between *β*-catenin and the androgen receptor that JNA develops virtually exclusively in men. JNA generates many blood vessels to support its growth, which makes it extremely vascularized. Researchers discovered that the tumor overproduces several growth factors that encourage the creation of blood vessels and the growth of tissue, such as VEGF, TGF-*β*1, and IGF-II.

Because of its propensity to bleed profusely during procedures, JNA is very difficult to remove surgically. Mutations in the *β*-catenin gene, however, seem to be widespread. Although some cases exhibit a lack of TP53 expression, the p53 tumor suppressor gene, which is commonly altered in malignancies, does not appear to play a significant role in JNA. Furthermore, the oncogenes c-myc and c-fos are frequently overexpressed, which may be a factor in the tumor’s quick development [1], 12].

Although surgery is still the mainstay of treatment, there is increasing interest in using complementary therapies, such as radiation therapy and targeted treatments, to overcome the drawbacks of surgery, especially when the tumor is large or involves hard-to-reach places. Treatments that focus on angiogenesis, or the development of new blood vessels, may be helpful because JNA is highly vascular. Anti-angiogenic drugs may lessen surgical bleeding or aid in tumor shrinkage. Hormonal treatments like estrogen or androgen blockers have also attracted attention, although their efficacy is still debatable [8], 9], 12].

As for other tumors [13], understanding the molecular pattern of JNA is crucial for proposing new therapeutic strategies. TNF-*α* contributes to the inflammatory and fibrotic reactions within the lesion, especially in high-grade JNA tumors [14]. Moreover, it enhances neoangiogenesis as well. These findings suggest the possible strategic value of TNF- TNF-*α*-targeted molecular therapies in JNA adjuvant treatments. VEGF is well-known to propel the construction of the vast vascular network of JNA [15], and along with TNF-*α*, can be found in high-grade JNA, confirming its pivotal role in promoting tumor growth. TGF-*β* is essential for stromal remodeling [16]. It is localized in the fibroblasts and endothelial cells within JNA tumors. This suggests a role in stimulating the proliferation of these cells, with higher density in more aggressive tumors [16].

The study aims to understand the molecular and histopathological mechanisms driving juvenile nasopharyngeal angiofibroma (JNA). It examines the function of TGF-*β*, VEGF, and TNF-*α* in angiogenesis, fibrosis, and inflammation – three important processes in the development of tumors – by examining tissue samples. The precise participation of these components is confirmed by comparing JNA with control tissues, which may assist direct the development of novel therapeutic approaches to slow tumor growth and surgical consequences.

## Materials and methods

### Clinical evaluation

Written informed consent concerning the donation of pediatric tumor tissues was provided by parents of the patients before tissue acquisition, following the protocol for the acquisition of human brain tissues of the Ethical Committee of the University Hospital Policlinico Umberto I, “Sapienza” University of Rome, Italy; which specifically approved the study protocol. All specimens were acquired in accordance with the principles of the Helsinki Declaration. Samples harvested for this study were obtained from pediatric patients admitted to the Department of Radiology, Oncology and Pathology, “Sapienza” University of Rome. Specimens consisted of 12 Nasopharyngeal Angiofibroma (8 males and 4 females, mean age 16.2, range 14–18 years) and 4 control samples (normal tissue samples contained no visible tumor cells; three males and one female) from archival paraffin embedded blocks with a sufficient sample size. The technique chosen for the immunohistochemical study of the samples was microdensitometry. Specifically, the choice of fragments to be studied focused on hot spots with the highest representation of tumor cells. The histopathological diagnosis of the 12 observed Nasopharyngeal Angiofibroma was confirmed by university pathologists. Of the 12 samples taken, 7 were grade II tumors, and the remaining 5 were grade III. None of the patients underwent embolization or radiotherapy before surgical treatment. Control samples were taken from the para-adenoid region in patients undergoing adenoidectomy; these microfragments of mucosa have been retrieved in patients with analogous demographic features of the 12 selected cases.

### Immunohistochemistry (IHC)

The immunohistochemical analysis was conducted using the ABC/HRP technique (avidin complexed with biotinylated peroxidase) on 4 µm thick paraffin sections that were cut using a rotative microtome. These sections were deparaffinized and hydrated through decreasing ethanol series to distilled water, then subjected to microwave irradiation and immersed in citrate buffer (pH=6) twice for 5 min each time. Subsequently, endogenous peroxidase activity was quenched using 0.3 % hydrogen peroxide in methanol for 30 min. To evaluate the immunolocalization of TGF-*β*1, VEGF-A, and TNF-*α* the following antibodies were employed: i) rabbit anti-TGF-*β*1 polyclonal antibody (Santa Cruz, CA, USA); ii) mouse anti-VEGF monoclonal antibody (Santa Cruz, CA, USA); iii) mouse anti-TNF-*α* monoclonal antibody (1:100, Santa Cruz Biotechnology). Overnight incubation with the primary antibodies was executed at 4 °C. Optimal antibody dilution and incubation times were assessed from data from preliminary experiments. As a negative control, the primary antibodies were omitted. After having exposed all slides to the primary antibodies, they were rinsed twice using a phosphate buffer (pH=7.4) and incubated for 1 h with the appropriate secondary biotinylated antibody at the final dilution of 1:200. The secondary biotinylated antibodies against rabbit and mouse immunoglobulins were provided by Abcam (biotinylated goat anti-rabbit antibody and biotinylated goat anti-mouse antibody). The slides were then incubated with peroxidase-conjugated avidin (Vector Laboratories, Burlingame, CA, USA, Vectastain Elite ABC kit Standard*PK 6–100) for 30 min. Slides were washed in phosphate buffer (pH=7.4) and treated with 0.05 % 3,3-diaminobenzidine (DAB) and 0.1 % H2O2. Sections were then counterstained using Mayer’s hematoxylin and dehydrated rapidly. Staining was performed by three experts. Microdensitometry was used to assess the intensity of the immune reaction (IAS 2000 image analyzer, Delta Sistemi, Rome, Italy) connected to the microscope using a TV camera. 12 100 µm^2^ areas were identified in each section by diaphragm measurements; the system was calibrated, setting the background obtained in non-immune serum-exposed sections as zero. Analysis of variance (ANOVA) followed by Duncan’s multiple range test as a post hoc test was used for quantitative data analysis of the intensity of immune staining. The expression levels of each antigen between pathological tissue and control tissue were compared using Student’s *t*-test.

### Ethics approval statement

All human studies have been reviewed by IRB and have therefore been performed in accordance with the ethical standards laid down in an appropriate version of the Declaration of Helsinki.

### Patient consent statement

Written informed consent has been acquired for every patient included in the present study. An original copy of a blank institutional consent can be provided upon request to the corresponding author

## Results

Microscopic examination of angiofibroma samples from patients between the ages of 14 and 18 showed a characteristic neoplastic growth with a dense fibrous stroma and complex vascular architecture. The vascular structures, which were all lined by a single layer of endothelial cells, showed significant variation in size and shape, ranging from huge lacunar-like voids to thin, elongated capillaries. Dense connective tissue and spindle-shaped or stellate fibroblasts, which were mainly distributed in a perivascular arrangement, made up most of the stroma. This fibrous background probably aids in the lesion’s mechanical stability and influences the growth of the tumor. Interestingly, amorphous foreign material was found in several arterial lumina, which may indicate previous embolization, a typical preoperative procedure for angiofibromas intended to lessen intraoperative hemorrhage. Given the homogeneous representation in terms of age and gender, these variables did not affect the significance of the results. Even concerning tumor grading, we did not find any particular differences between type II and type III tumors. As for the possible impact of embolization treatment, it was not possible to include this parameter in the analysis because no patients underwent neoadjuvant treatments before surgery.

### Immunohistochemical findings

#### TGF-β expression and its role in extracellular matrix remodeling

**Figure 1:**
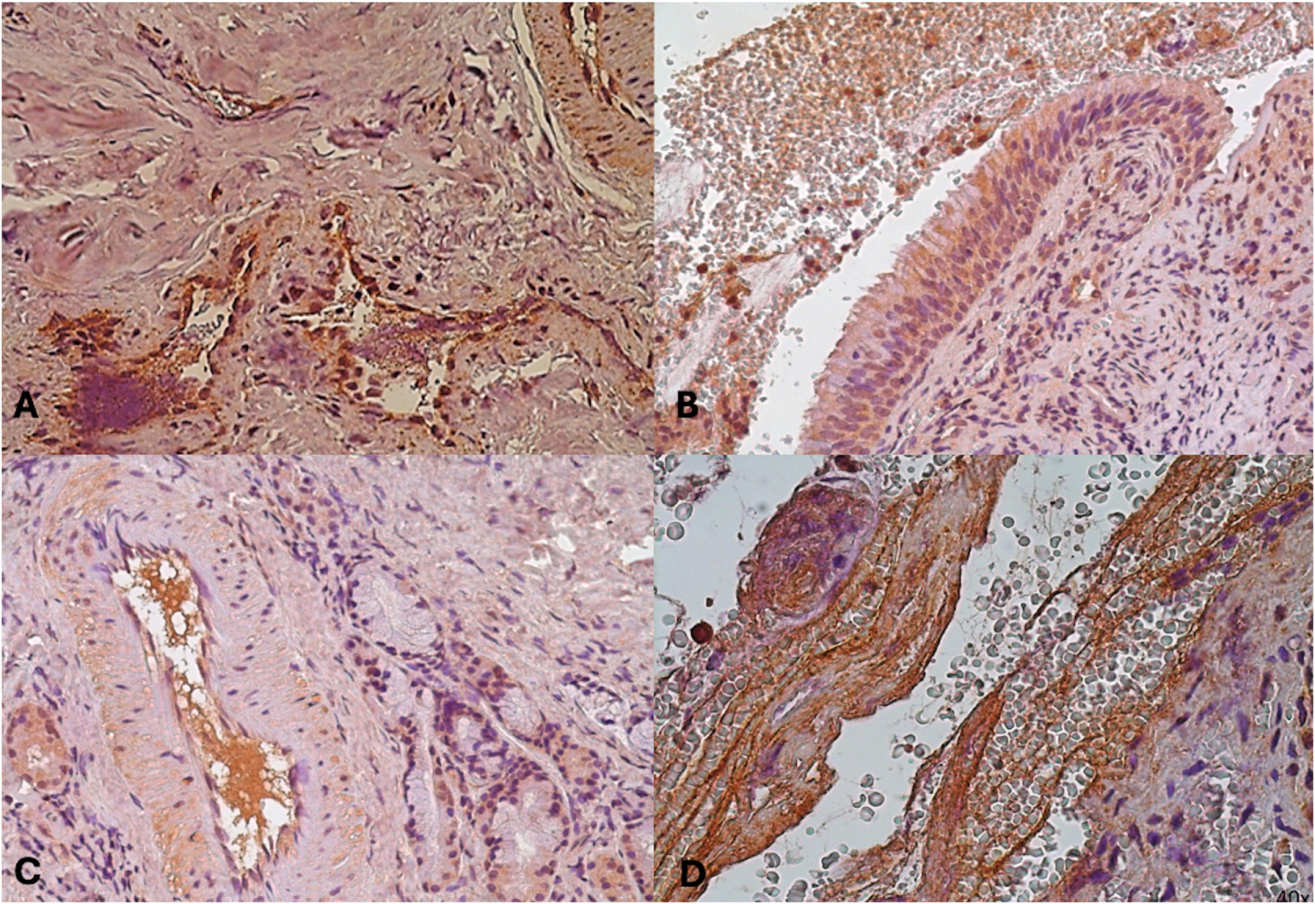
Expression of growth factor TGF-*β in different cases*. (A) Immunohistochemical expression of growth factor TGF-*β* (20x). 16 years old. (B) Immunohistochemical expression of growth factor TGF-*β* (20x). 16 years old. (C) Immunohistochemical expression of growth factor TGF-*β* (20x). 18 years old (D) immunohistochemical expression of growth factor TGF-*β* (40x). 14 years old.

**Figure 2:**
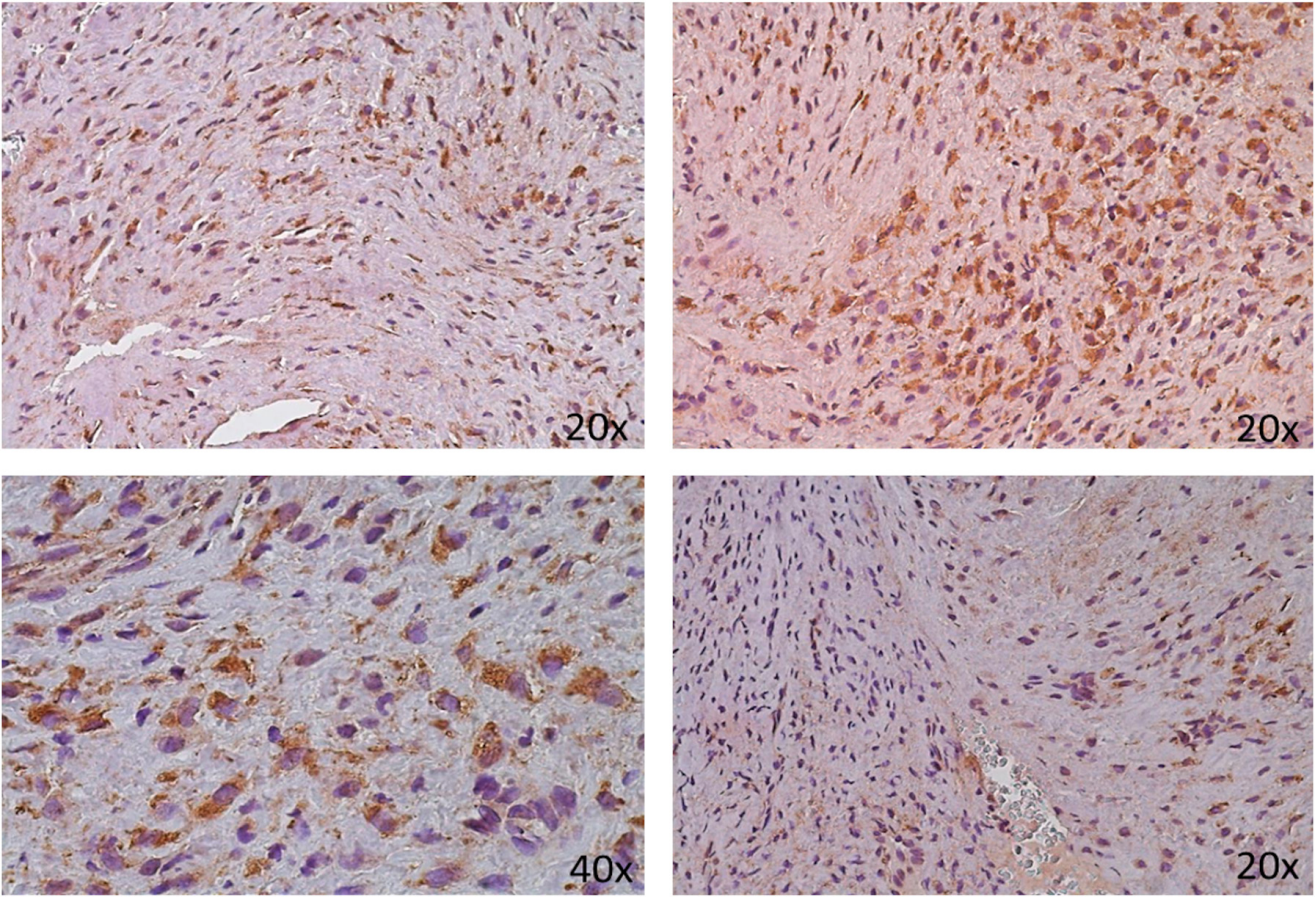
Immunohistochemical expression of growth factor TGF-*β*. 14 years old.

The tumor vasculature’s endothelial cells showed high expression of Transforming Growth Factor Beta (TGF-*β*) (Figures 1 and 2). The neoformation seems to be made up of a single layer of endothelial cells lining a proliferation of vascular structures of varying caliber, which are now filiform and lacunar in appearance. Spindle or star-shaped fibroblasts are primarily seen in a perivascular arrangement within the largely dense fibrous connective stroma that surrounds these arteries. There are noticeable globules of amorphous foreign material inside the vessels, which are probably related to embolization (Figure 1).

High-magnification (40×) examination of certain samples showed clear cytoplasmic positivity, which suggests active intracellular signaling. (Figure 1D, 2). TGF-*β* expression was found in the extracellular matrix (ECM) in addition to endothelial cells, and it was linked to a rise in myofibroblast proliferation there (Figure 2). Figure 2D shows neoplastic growth with hypocellular fibrous tissue around many arterial channels and high expression of TGF at the level of vascular endothelium, together with significant cytoplasmic positivity of this cytokine, in a 14-year-old subject.

These findings suggest that TGF-*β* plays a crucial role in the fibrotic character of the stroma by promoting the alteration and deposition of extracellular matrix (ECM), which may contribute to the growth of tumors by creating an environment that supports the formation of new blood vessels.

#### VEGF overexpression and its angiogenic implications

**Figure 3:**
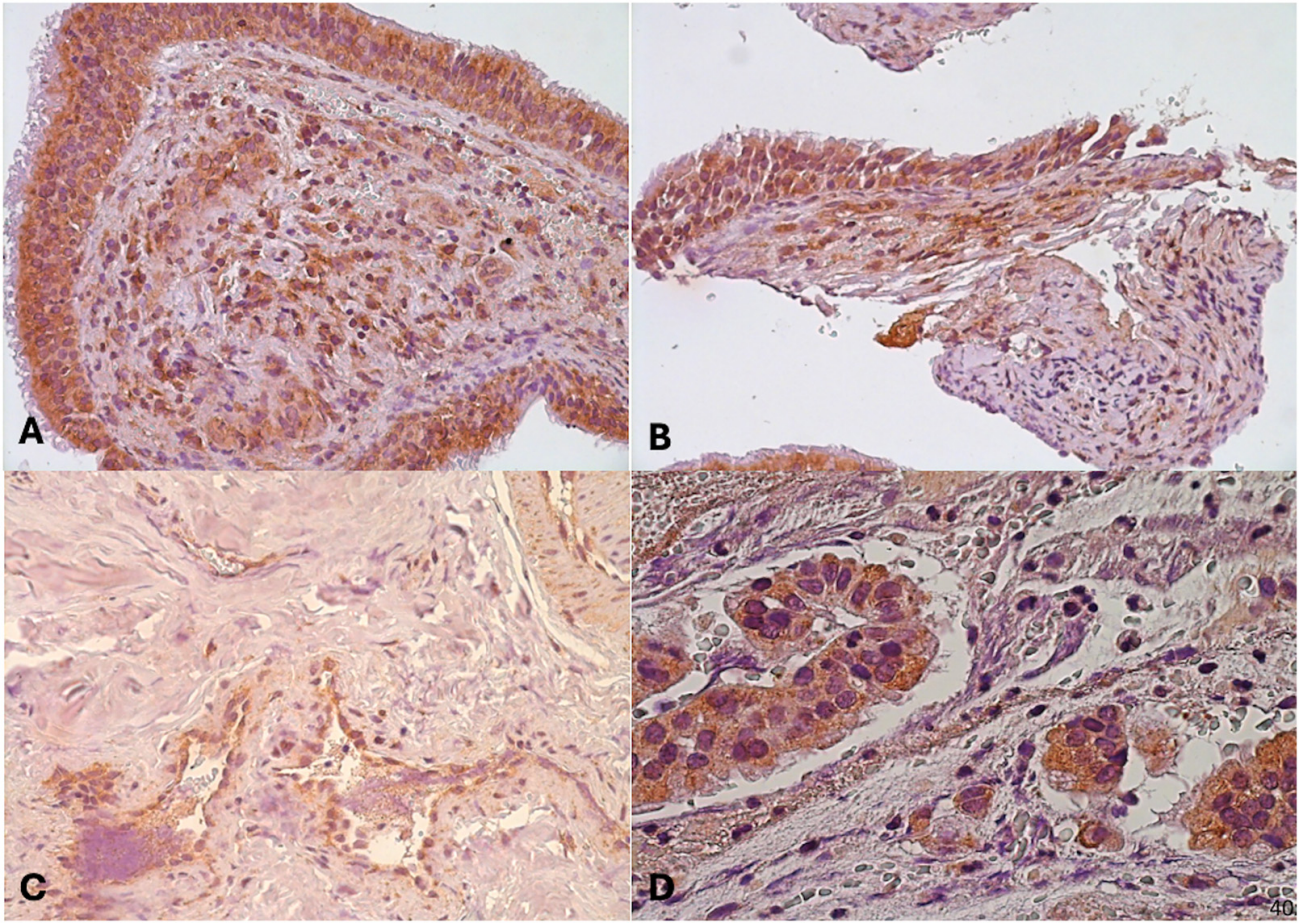
Expression of VEGF growth factor *in different cases*. (A) Immunohistochemical expression of VEGF growth factor (20x). 14 years old. (B) Immunohistochemical expression of VEGF growth factor (20x). 14 years old. (C) Immunohistochemical expression of VEGF growth factor (40x). 14 years old. (D) Immunohistochemical expression of VEGF growth factor (40x). 14 years old.

The high immunoreactivity of vascular endothelial growth factor (VEGF) within endothelial cells supports its function in tumor-driven angiogenesis (Figure 3). Furthermore, VEGF was expressed in the glandular epithelium of certain specimens, indicating a function in local tissue vascularization that goes beyond endothelial proliferation, as shown in Figure 3 where a respiratory-type mucosa with neoplastic proliferation consisting of hypocellular fibrous tissue circumscribing numerous vascular channels is evident. Since it promotes endothelial cell migration, proliferation, and survival, VEGF overexpression is a well-established mechanism in the pathophysiology of angiofibromas and ultimately contributes to the highly vascular nature of the tumor. The clinical finding that angiofibromas are hypervascular lesions that are vulnerable to severe intraoperative hemorrhage is consistent with the extensive VEGF immunostaining.

#### TNF-*α* positivity and its inflammatory contribution

**Figure 4:**
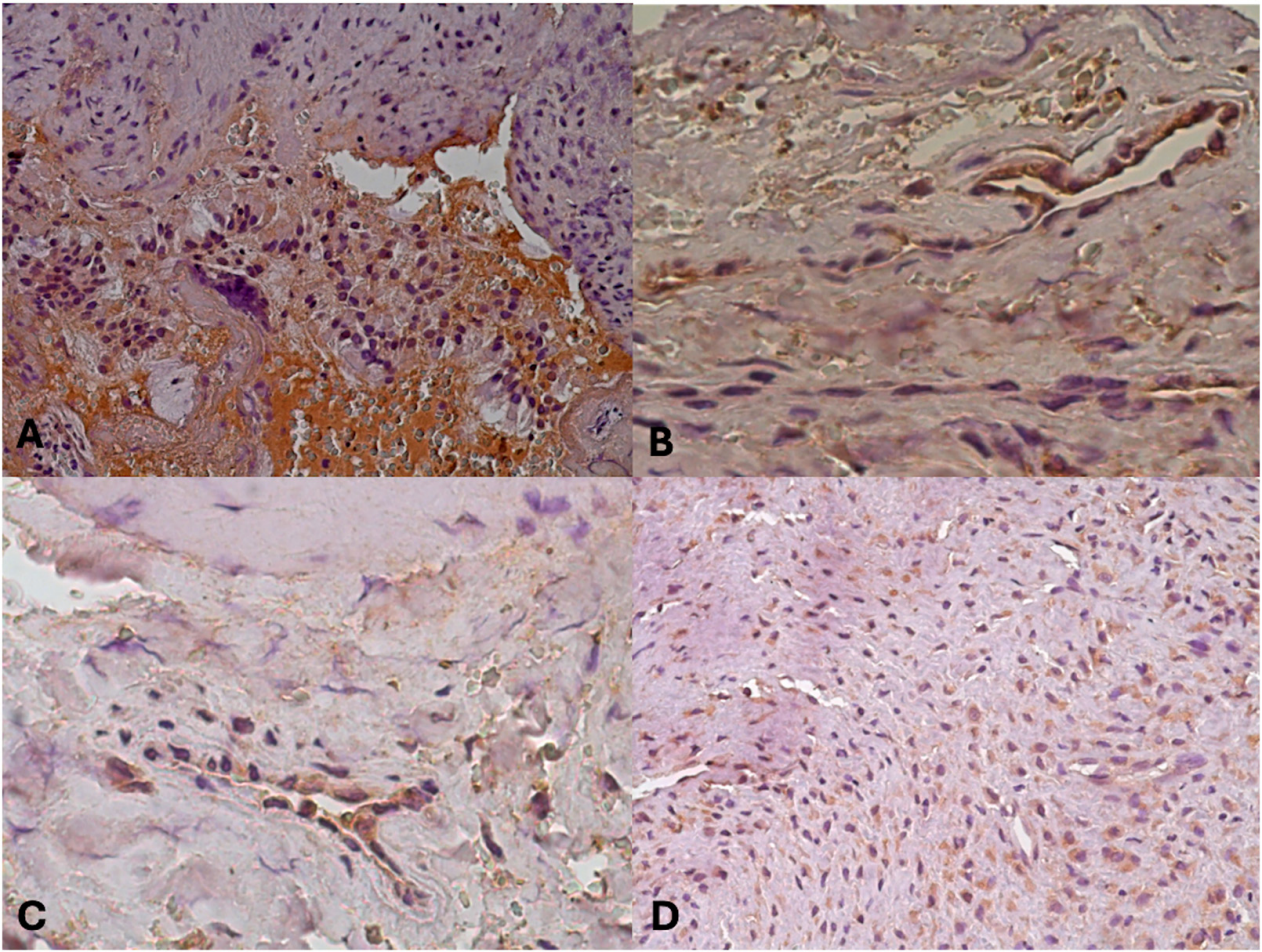
Expression of growth factor TNF-*α* *in different cases. *(A) Expression of growth factor TNF-*α* (20x). 14 years old. (B) Immunohistochemical expression of growth factor TNF-*α* (40x). 14 years old. (C) Immunohistochemical expression of growth factor TNF-*α* (40x). 16 years old. (D) Immunohistochemical expression of growth factor TNF-*α* (20x). 14 years old.

In several samples, Tumor Necrosis Factor Alpha (TNF-*α*) demonstrated significant cytoplasmic immunoreactivity, with several displaying strong positivity (Figure 4). Figure 4A shows a respiratory-type mucosa with neoplastic proliferation consisting of hypocellular fibrous tissue and cytoplasmic immunoreactivity for TNF-*α*. This implies that the pro-inflammatory environment of angiofibromas may be influenced by TNF-*α*. TNF-*α* may help stromal remodeling and encourage the persistent inflammation frequently seen in these lesions because of its established function in controlling cell survival, apoptosis, and fibrosis. Additionally, it may improve tumor vascularization and fibrosis through its interaction with the VEGF and TGF-*β* pathways, creating a feed-forward loop that maintains tumor growth.

### Comparison with control samples

**Figure 5:**
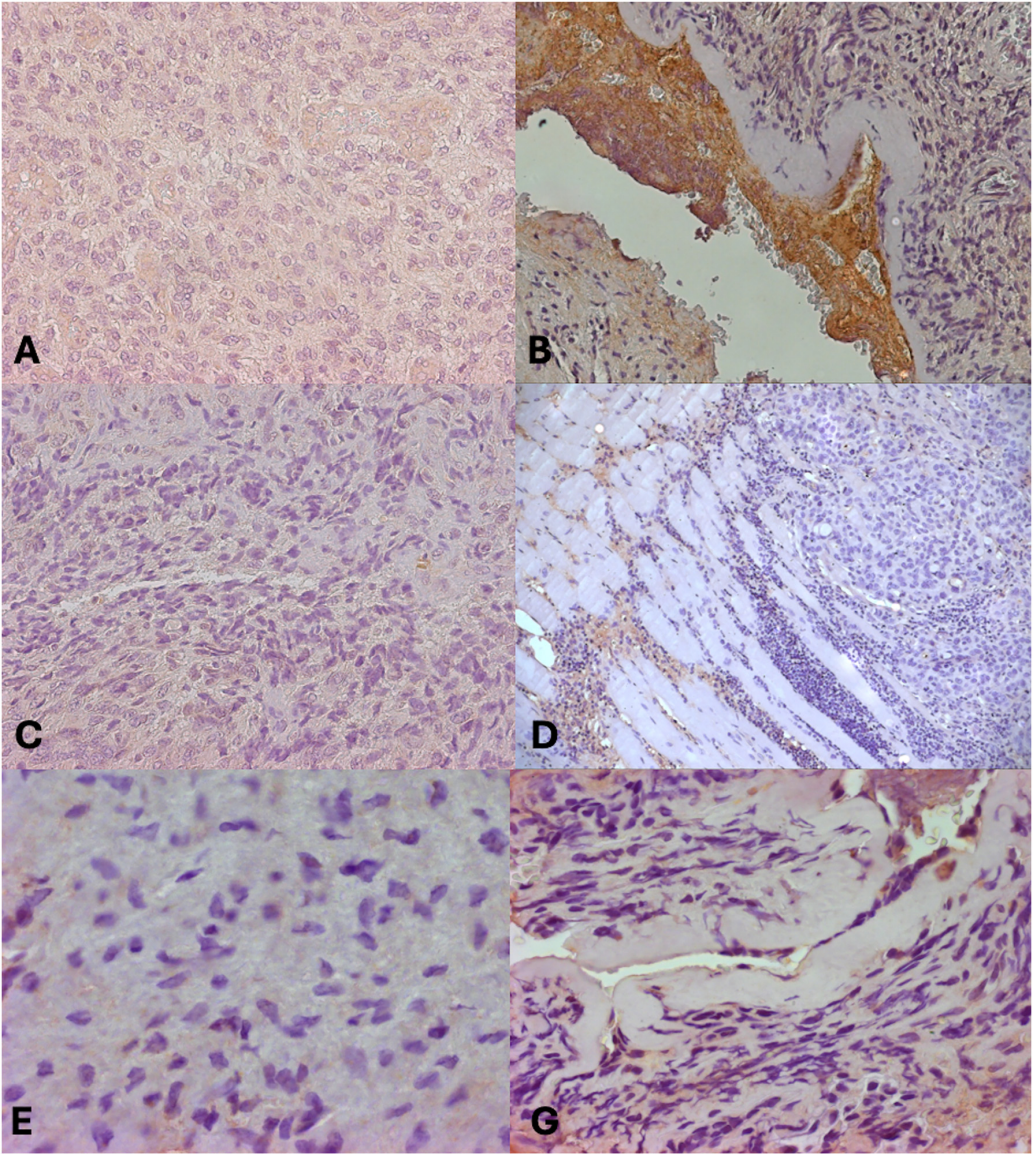
Expression of VEGF, TGF-*β* and TNF-*α* growth factors in case controls. (A) Immunohistochemical expression of VEGF growth factor (20x). Case control. (B) Immunohistochemical expression of VEGF growth factor (10x). Case control. (C) Immunohistochemical expression of growth factor TGF-*β* (20x). Case control. (D) Immunohistochemical expression of growth factor TGF-*β* (10x). Case control. (E) Immunohistochemical expression of growth factor TNF-*α* (40x). Case control. (F) Immunohistochemical expression of growth factor TNF-*α* (40x). Case control.

Control samples (Figure 5) that were stained for TNF-*α*, VEGF, and TGF-*β* showed little to no immunoreactivity, indicating that the staining patterns seen in angiofibroma tissues were particular. Instead of being coincidental observations, this supports the theory that these growth factors actively contribute to the pathophysiology of the tumor.

### Limitations

In light of what has been said so far, it is important to note that one limitation of this study is the small size of the sample considered. Therefore, although the results are suggestive, some data, such as tumor grading and the evaluation of neoadjuvant treatments, should be considered with caution and will need to be studied in depth with larger cohorts.

## Conclusions

Histopathological and immunohistochemical results lend credence to the idea that a complicated interaction between fibrotic and angiogenic components drives the formation of angiofibromas. TNF-*α* contributes to the inflammatory and fibrotic reactions within the lesion, VEGF propels the construction of the vast vascular network, and TGF-*β* is essential for stromal remodeling. These molecular discoveries not only improve our comprehension of the pathophysiology of angiofibroma but also lay the groundwork for the creation of innovative treatment approaches [12], [17], [18], [19], [20], [21], [22], [23], [24].

Alternative therapeutic strategies targeting these molecular pathways are showing increasing interest because of the difficulties of surgical resection, mainly caused by hypervascularization of the tumor and likely invasion into contiguous anatomic tissues.

Anti-angiogenic treatments that target VEGF signaling have been developed because VEGF plays a major role in the growth of JNA tumors. Although their efficacy has been limited, these treatments have been investigated as adjuvant possibilities for highly vascular JNA cases, especially in difficult surgical placements [25]. VEGF, TGF-*β*, or TNF-*α* inhibitors may present viable non-surgical or neoadjuvant alternatives to lessen tumor vascularization and fibrosis, lowering surgical morbidity or, in some situations, acting as stand-alone therapies. New treatments, such as small-molecule inhibitors and tailored monoclonal antibodies, like the VEGF-antibody Bevacizumab [26] may help slow the growth of tumors while lowering the dangers connected with drastic surgical procedures [1], 6], 8], 18], 27].

For refractory instances, other molecular targets such as insulin-like growth factors (IGFs) and TGF-*β*1 have the potential to be used as neoadjuvant therapy. Furthermore, studies on the Wnt/*β*-catenin pathway might offer other treatment options. Genetic screening for familial adenomatous polyposis (FAP) should be taken into consideration in JNA patients due to the potential association between JNA and FAP [18], 23].

Some studies have also evaluated the effect of the antineoplastic anti-androgen drug flutamide in an attempt to minimize intraoperative blood loss and reduce tumor volume, allowing surgical excision by more conservative procedural methods. It appeared most effective in the early stages of JNA without significant tumor invasion but could also be useful as a neoadjuvant drug in some patients, reducing tumor volume before excision with greater tumor volume reduction in postpubertal patients [26], [27], [28].

The effectiveness and safety of these innovative treatments need to be confirmed by more research, which could revolutionize angiofibroma treatment and enhance patient outcomes.
